# Serum profiling of healthy aging identifies phospho- and sphingolipid species as markers of human longevity

**DOI:** 10.18632/aging.100630

**Published:** 2014-01-21

**Authors:** Ivan Montoliu, Max Scherer, Fiona Beguelin, Laeticia DaSilva, Daniela Mari, Stefano Salvioli, François-Pierre J. Martin, Miriam Capri, Laura Bucci, Rita Ostan, Paolo Garagnani, Daniela Monti, Elena Biagi, Patrizia Brigidi, Martin Kussmann, Serge Rezzi, Claudio Franceschi, Sebastiano Collino

**Affiliations:** ^1^ NESTEC SA, Nestlé Research Center, Vers-chez-les-Blanc, CH-1000 Lausanne 26, Switzerland; ^2^ Nestlé Institute of Health Sciences SA, Campus EPFL, Molecular Biomarkers Core, Quartier de l'innovation, CH-1015 Lausanne, Switzerland; ^3^ Department of Medical Sciences, University of Milan, Milan, Italy; ^4^ Geriatric Unit IRCCS Ca' Grande Foundation Maggiore Policlinico Hospital, Milan, Italy; ^5^ Department of Experimental, Diagnostic and Specialty Medicine Experimental Pathology, University of Bologna, Via S. Giacomo 12, 40126 Bologna, Italy; ^6^ Interdepartmental Centre “L. Galvani” CIG, University of Bologna, Piazza di Porta S. Donato 1, 40126 Bologna, Italy; ^7^ Department of Clinical, Experimental and Biomedical Sciences, University of Florence, Viale Morgagni 50, 50134 Florence, Italy; ^8^ Department of Pharmaceutical Sciences, University of Bologna, Via Belmeloro 6, 40126 Bologna, Italy; ^9^ Faculty of Life Sciences, Ecole Polytechnique Fédérale Lausanne (EPFL), Lausanne, Switzerland; ^10^ Faculty of Science, Interdisciplinary NanoScience Center (iNANO), Aarhus University, Aarhus, Denmark

**Keywords:** Healthy Aging, metabolomics, lipidomics, biomarkers, inflammageing

## Abstract

As centenarians well represent the model of healthy aging, there are many important implications in revealing the underlying molecular mechanisms behind such successful aging. By combining NMR metabonomics and shot-gun lipidomics in serum we analyzed metabolome and lipidome composition of a group of centenarians with respect to elderly individuals. Specifically, NMR metabonomics profiling of serum revealed that centenarians are characterized by a metabolic phenotype distinct from that of elderly subjects, in particular regarding amino acids and lipid species. Shot- gun lipidomics approach displays unique changes in lipids biosynthesis in centenarians, with 41 differently abundant lipid species with respect to elderly subjects. These findings reveal phospho/sphingolipids as putative markers and biological modulators of healthy aging, in humans. Considering the particular actions of these metabolites, these data are suggestive of a better counteractive antioxidant capacity and a well-developed membrane lipid remodelling process in the healthy aging phenotype.

## INTRODUCTION

Population aging has emerged as a major demographic trend worldwide due to improved health and longevity. This global aging phenomenon will have a major impact on health-care systems worldwide due to increased morbidity and greater needs for hospitalization/institu-tionalization. As the life expectancy of population increases worldwide, there is an increasing awareness of the importance of “healthy aging” and “quality of life”. Aging is a very complex process since several alterations of biochemical processes occur during the entire course of life, affecting all levels, from organs to cells. Indeed, aging appears to be characterized by chronic, low-grade systemic inflammation, a condition indicated as inflammaging [1-2], that is believed to contribute to the onset and progression of the major age-related diseases, also including frailty [3-5] and for the most of deaths in elderly. However, this process is not completely understood. In particular, centenarians display signs of inflammaging but at the same time seem to be spared from its deleterious consequences. It is hypothesized that this apparent paradox is explained by the fact that they possess a complex and peculiar balancing between pro-inflammatory and anti-inflammatory factors, resulting in a slower, more limited and balanced development of inflammaging, in comparison with old subjects, who are characterized by either faster or inadequately counteracted inflammatory responses [6]. A large amount of data indicates that inflammation is strictly connected to oxidative stress. Both inflammation and oxidative stress can deeply affect a number of metabolic processes and therefore is to be expected that many differences exist on metabolome of centenarians that can be helpful in shedding light on the mechanisms of successful aging.

Systems-level omics technologies are emerging as a valuable approach to comprehensively investigate changes in metabolic regulations, and then linking these to the phenotypic outcome [7-9]. Metabolomics, the quantitative profile of endogenous and exogenous small molecules present in biological systems, had successfully been applied to study the modulation of the aging processes following nutritional interventions, including caloric restriction in mouse [10], dogs [11], and non-human primates [12]. In order to better elucidate molecular mechanisms that involve the disruption of lipid metabolic pathways, the field of lipidomics is also being rapidly emerging. Lipidomics can be achieved by a comprehensive measurement of the lipidome, i.e. the complete set of biological lipids, from a single analysis in a non-targeted profiling way (shotgun approach) [13]. An investigation on specific lipids associated with familial longevity in females was explored in the plasma lipidome by comparing lipid species in offspring of nonagenarian vs. elderly controls from the Leiden Longevity Study [14], starting from the assumption that a heritable component of the lipidome profile typical of longevity should exist. In fact, this study identified in women but not in men nineteen lipid species that resulted significantly different in nonagenarian offspring and age-matched controls, with ether phosphocholines and sphingomyeline species identified as putative longevity markers. Nevertheless in this study there are no data on subjects who reached extreme longevity, such as centenarians (mean age of the subjects was around 60 years), and the possibility exists that the differences observed were due to an altered health status, as an association of some of these markers with hypertension and Type 2 Diabetes was reported.

We originally exploited a different experimental model that included centenarians, a recognized model of successful aging [1,2,15], as well as old people and younger controls to unveil the metabolic phenotype of aging and longevity, and we observed that centenarians possess a unique metabolic phenotype characterized by differential eicosanoids network and higher concentrations of bacterial metabolism by-products [15]. In the present study, we went further by investigating metabolomics, and specific lipidomics changes in blood serum of centenarian' subjects, to identify candidate metabolic signatures of healthy aging. Centenarians were on average aged 101 years, ±2, while the group of elderly individuals was on average aged 70 years±6. All subjects were recruited in Northern Italy.

## RESULTS

### Serum ^1^H-NMR profiles display amino acids and lipid metabolism as metabolic signatures of longevity

**Figure 1:**
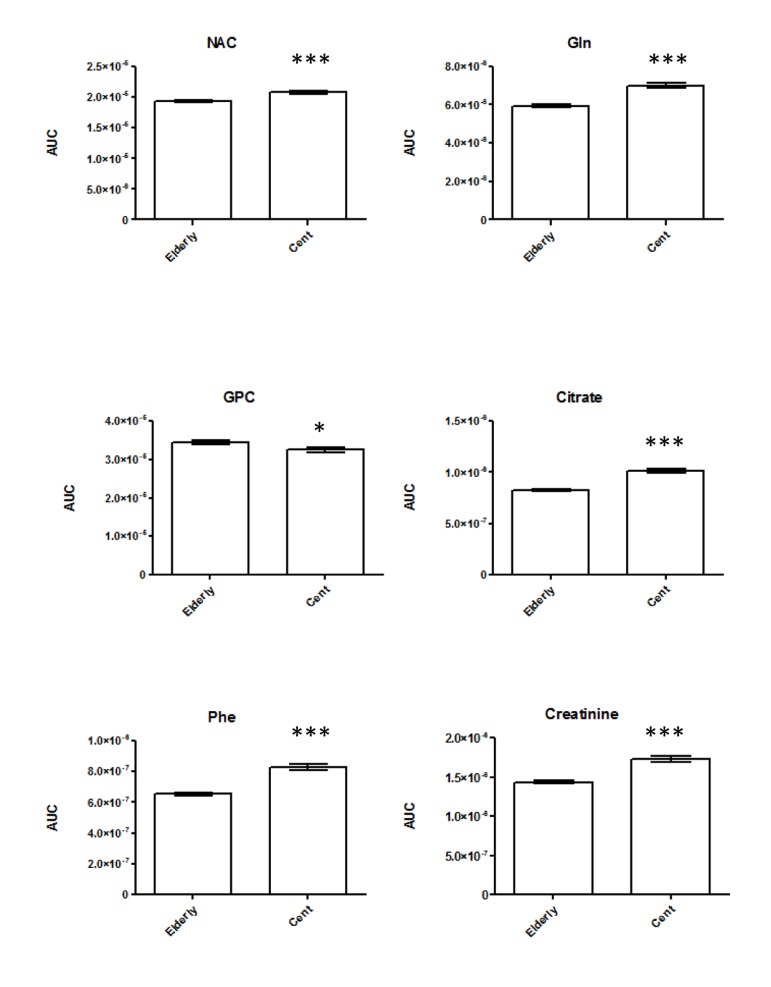
Bar plots representing differences in serum markers of longevity as per ^1^H-NMR. All significantly regulated metabolites and statistical changes are listed in Table S2.

For NMR metabolomics we focused on analysis of CPMG spectral data rather than standard 1D ^1^H-NMR spectroscopic data, in order to characterize the differences in signals from low molecular weight metabolites that may be overlapped by broad signals from proteins and lipoproteins. 600 MHz ^1^H-NMR Carr−Purcell−Meiboom−Gill sequence (CPMG) metabolic profiling of serum samples was performed on 98 centenarians and 196 elderly (Table 1) to specifically reveal low molecular weight metabolites. To explore age- induced changes and metabolic differences among the two age groups and minimize any effects of non-relevant metabolite variability, supervised chemometric analysis of the serum NMR profiling was applied on the full resolution NMR data set. Orthogonal Projection on Latent Structures – Discriminant Analysis (OPLS-DA) was carried out on unit variance scaled data. As previously published [15] the discriminant model between centenarians and elderly group provided a validation error of the classifier (expressed as area under the ROC curve, AuROC) of 0.99 by using a 15.4% of the spectral variance (R^2^X) (Table S1). A clear separation was achieved between the ^1^H-NMR spectra of the elderly and from centenarians (Figure S1A-S1B), indicating that long-lived subjects are characterized by a separate metabolic phenotype. To determine the metabolic signatures associated to the differences between age groups, loadings of the first predictive component of the OPLS-DA model were used, color coded according to the correlation coefficient of the variable [16] (Fig. S1). Here, the colors projected onto the coefficient plot indicate the correlation of the intensities of the peaks from the metabolites discriminating centenarians from the corresponding elderly. Accordingly, the serum discriminant model display in centenarians relatively lower amount of glycerophosphocholine (GPC), and higher amounts of N-acetlyglycoproteins (NAC), glutamine, citrate, creatinine, and phenylalanine. To gain semi-quantitative information, peak areas in the original spectra were integrated for these metabolites (Figure 1) and differences with statistical significance were confirmed by paired t tests (2 tailed)(Table S2). As the majority of centenarians are females, it is unworthy to perform gender separation as this leads to limited statistical power. However for qualitative indication we report values for females and males (Tables S3-S4), displaying that the overall above trend is kept.

**Table 1:** Clinical characteristics of the aging cohort. Values are presented as mean (±SD) with the range in parentheses. P value is as follow: *p<0.05, **p<0.01, ***p<0.001.

	Centenarians	Elderly
Gender, *male/female* Age, years	22/76 100.7^±^2	85/111 70.4^±^6
BMI, *kg/m^2^*	24.0^±^3.8 (17.8-29.2)	26.8^±^1.5 (16.1-49.7)**
HOMA *index*	1.97^±^0.66 (0.3-2.1)	3.0^±^2.8 (0.7-16.4)*
Cholesterol, *mg/dl*	185.0^±^32.7 (112-264)	201.0^±^37.2 (5-335)**
Triglycerides, *mg/dl*	114.4^±^46.1 (60-283)	129.9^±^65.7 (44-530)***
HDL-cholesterol, *mg/dl*	48.2^±^13.1 (25-99)	55.2^+^20.4 (20-147)*
LDL- cholesterol, *mg/dl*	105.6^±^35.1 (75-165	118.7^±^45.7 (23.8-199)*
CRP, mg/L	5.41^±^4.9 (0.28-19.9)	3.24^±^3.9 (0.11-19.1)*
A-SAA, *μg/ml*	527^±^707 (15.5-3821)	142.9^±^187 (0.01-1318)**
MMSE^1^	21.2^±^4.2 (15.2-23.2)	27.16±1.53 (23-29.7)***
Cardiovascular therapy, *%*	33	67
Irregular heart rhythm, *%*	14	21
Diabetes^2^, *%*	5	11

Legend: BMI=body mass index, HOMA=Homeostatic Model Assessment index, HDL= high density lipoprotein, LDL= low density lipoprotein,CRP=C reactive protein, A-SAA= Serum amyloid A (SAA) proteins.

1 MMSE= Cognitive function measure using the Mini-Mental State Examination (MMSE). The score used in the analysis was corrected by age and years of educations according to Magni et. al for old people. MMSE for elderly cognitive impairment was graded as severe (score 0–17), mild (score 18–23), or not present (score 24–30). MMSE for centenarians ≥ 20 absence of severe cognitive decline; <12 presence of severe cognitive decline according to Franceschi et al.2000a.

2 Diabetes mellitus: history of diabetes, fasting glucose plasma ≥126mg/dl

### Shot-gun lipidomics identify markers of longevity in serum

**Figure 2:**
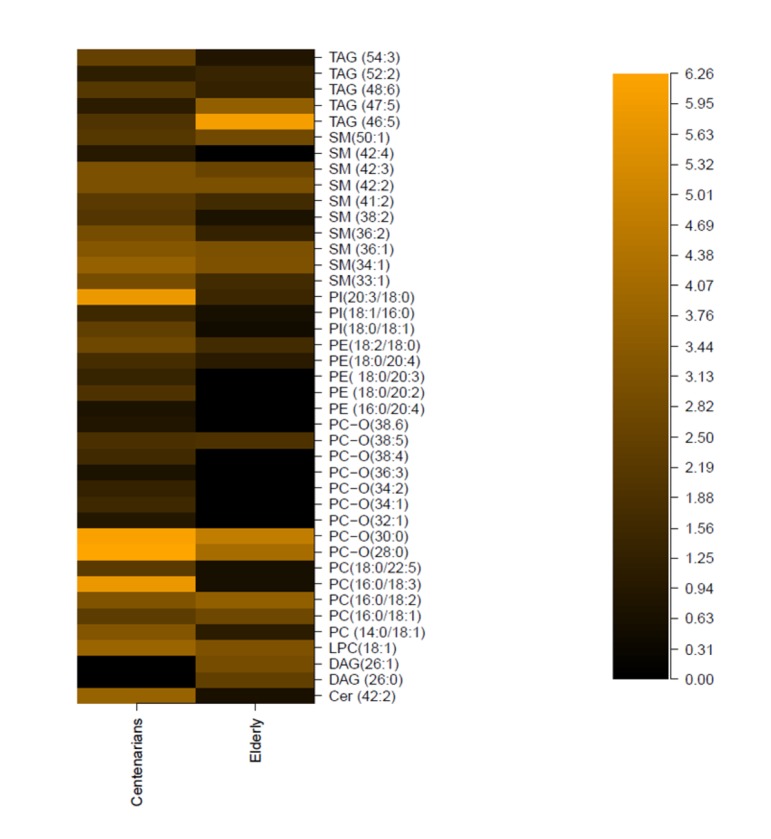
Differences in mean (μM) standardized lipids value between elderly and centenarians. Represented is median value (μM) of each metabolite divided it by the IQR range of the distribution. All significantly regulated metabolites are listed in Table S5.

A MS/MS shot gun lipidomics approach was applied on serum samples from 15 centenarians and 37 elderly. Multivariate data analysis was performed using Random Forests (RF™) [15] on quantitative data from thirteen different lipid classes: triacylglycerol TG (n=30), sphingomyelin SM (n=25), lysophosphatidylcholine LPC (n=7), phosphatidylcholine PC (n=34), ether phosphatidylcholine PC-O (n=19), ceramides Cer (n=6), phosphatidylethanolamine PE (n=14), phosphatidylethanolamine based ether PE-O (n=9), lyso-phosphatidylethanolamine LPE (n=3), phosphatidylinositol PI (n=7), phosphatidic acid PA (n=1), diacylglycerol DAG (n=19). Using the variable importance feature implemented in RF™, it was possible to determine the metabolic signature that better discriminates among elderly and centenarians. To assess the individual discriminant ability of each component of the signature paired t tests (2 tailed) was performed (all significantly regulated metabolites are listed in Table S5). Compared to elderly (Figure 2, table S5), relative concentration of sphingolipids increased in centenarians (Cer 42:2, SM 33:1, SM 34:1, SM 36:1, SM 36:2, SM 38:2, SM 41:2, SM 42:2, SM 42:3, SM 33:1, SM 42:4) glycerolphospholipids levels varies for selected species (increased in LPC 18:1, PC 14:0/18:1, PC 16:0/18:1, PC 16:0/18:2, PC 14:0/18:2, PC 16:0/18:3, PC 18:0/22:5; decreased in saturated PC-O 28:0, PC-O 30:0, increased in polyunsaturated PC-O 32:1, PC-O 34:1, PC-O 34:2, PC-O 36:3, PC-O 32:1, PC-O 38:4, PC-O 38:5, PC-O 38:6; increased in PE 16:0/20:4, PE 18:0/20:2, PE:18: 0/20:3, PE 18:0/20:4; increase in PI 18:0/18:1, PI 18:1/16:0, PI 20:3/18:0; increased in SM 33:1, SM 34:1, SM 36:1, SM 36:2, SM 38:2, SM 41:2, SM 42:2, SM 42:3, SM 42:4, SM 50:), and glycerol lipids increased/decreased (decreased in TG 46:5, TG 47:5, DAG 26:0, DAG 26:1, increased in TG 48:6, TG 52:2, TG 54:3). As in the case of ^1^H-NMR profiling, the majority of centenarians are female individuals, therefore performing gender separation leads to limited statistical power. However for qualitative indication we report values for females and males (Tables S6-S7), displaying the overall trend is kept. Classical clinical parameters display that our centenarian cohort has very low incidence of diabetes, and anthropometric (BMI), metabolic (cholesterol, LDL-C, HDL-C, triglycerides) values lower with respect to the elderly cohort. In addition, their cognitive function, measured using the Mini-Mental State Examination (MMSE).

## DISCUSSION

In the present study we have expanded our previous investigation on metabolic signatures of longevity by integrating a system biology approach in serum on a representative North Italian cohort of aged subjects, compromising elderly and centenarians. Data on centenarians in particular are of interest, as they are considered the best example of successful aging having reached the very extremes of the human lifespan. It is worth noting that the metabolic signatures described in this paper reflect mostly female individuals, as male centenarians are much more rare than female ones, and in particular in the north of Italy the male/female ratio is about 1:7 [17]. Therefore, our findings will be compared and further validated in much bigger cohorts to provide future predictive utility.

Metabolomics data display changes in amino acid metabolism with higher phenylalanine (Phe) concentrations in longevity. Phe is converted to tyrosine by the catalysis of phenylalanine hydroxylase [18]. It exhibits anti-inflammatory properties and it is often use to treat arthritis and Parkinson's disease [19]. We also noted an increase in glutamine in centenarians, which directly crosses the blood-brain barrier. This amino acid operates as a nitrogen shuttle, taking up excess ammonia and forming urea, therefore reducing toxic build up in the brain and improving brain functions [20-23]. Studies have also demonstrated that glutamine may play an important role on *NF-κB* signal transduction pathways, contributing to the attenuation of local inflammation [24]. The tricarboxylic acid (TCA) intermediate citrate, which may also play a role in amino acid and fatty acid metabolism, is also elevated in centenarians, relative to elderly. Interestingly, elevated plasma citrate levels inhibit phosphofructokinase, a regulatory enzyme in glycolysis, and glycolytic inhibitors such as 2-deoxyglucose have been put forward as viable dietary restriction (DR) mimetics [25]. Previous data on centenarians displayed high circulating levels of proinflammatory molecules [26]. Our NMR data display increase concentration of N-acetyl resonance of glycoproteins (NAC) [27], which are predominantly synthesized in liver parenchymal cells in response to cytokines [28]. Not surprisingly, serum creatinine concentration increased steadily with age [29], which is mainly produced by the metabolism of creatine in the muscle. Our metabolomics data also demonstrated decreased levels of the membrane metabolites glycerophosphorylcholine (GPC), in centenarians. Changes in GPC in longevity are of particular importance as senescent cells showed an increased concentration of this metabolite [30]. Elevated plasma levels of phospholipids and TMA were shown to be both risk factors for cardiovascular disease in humans [31;32]. Choline is also involved in synthesizing sphingomyelin and phosphatidylcholine, which are precursors for diacylglycerols and ceramides. In order to better assess changes in lipid metabolism we deployed a shot-gun lipidomics approach. We observed that centenarians display an overall increase in SM, which are important cellular messengers, with their low level associated to neurodegenerative diseases [33], atherosclerosis [34], and cardiovascular disease [35]. Among the ten SM whose levels are higher in centenarians, three species are of particular interest; SM 41:2, SM 36:2, SM 34:1. These have previously been suggested to represent lipidomic signatures of familial longevity in females [14]. SM can be converted to ceramides by the enzymatic activities of sphingomyelinases (SMases). It was suggested that SMases activity increase with age [36], therefore increasing ceramide contents, with their accumulation negatively effecting pro-inflammatory pathologies [37;38]. In atherogenesis, for example, ceramide accumulation is linked to aggregation of LDL, increased ROS, and promotion of foam cell formations [39]. Our data reflect that among the six measured ceramide only one (Cer 42:2) increases. An imbalance between the production of oxidants and protective antioxidant systems in favour of an excessive accumulation of reactive oxygen species (ROS) may cause cellular oxidative damage to nucleic acids and proteins in cells of several systems including the endocrine [40] and the immune [41]. Better antioxidant capacity/handling is confirmed by overall increase in centenarians of plasmalogen (PC-O) species, able to prevent oxidation of lipoproteins and cardioprotective [42]. Our study confirms increase of two ether phospholipids, PC-O 36:6 and PC-O 34:1, whose concentration was found to change in familial longevity [14], and one ether PC-O 32:1, previously determined in different elderly individuals, as metabolic marker of healthy aging [15]. Interestingly, conservation of membrane composition/integrity is also confirmed by the joined increase in phosphatidylcholine (PC), major components of cell membranes and phosphate-dylethanolamine (PE), a key modulator of inflammatory reaction in centenarians. Previously it was demonstrated that PC/PE ratio (i.e., increase of PC/decrease of PE and vice versa) is a critical modulator of membrane integrity [43]. Changes in the phospholipids distribution influence membrane protein function, modifying the permeability of solutes across the membrane [44] through changes in the fluidity of the bi-layer. Measurement of the fatty acid composition of human erythrocyte membrane lipids has shown that centenarians have a reduced susceptibility to peroxidative membrane damage, while higher membrane fluidity compared with all the other age groups [45]. In particular, the increase in PE is interesting as it was previously postulated that highly polyunsaturated PE can carry pro-inflammatory molecules such as the arachidonic acid lipid network [46]. Another phospholipid, phosphatidylinositol (PI), possesses immunoregulatory capacities [47]. Here we detect an increase in three PI species (PI 18:0/18:1, PI 18:1/16:0, PI 20:3/18:0) in centenarians. In animal tissues, phosphatidylinositol is the primary source of the arachidonic acid required for biosynthesis of eicosanoids, including prostaglandins, via the action of the enzyme phospholipase A2. We have previously displayed that centenarians possess a unique balanced of anti- and pro-inflammatory eicosanoids, therefore we believed that an increase in PE and PI mirrors these findings, displaying that centenarians possess an unique and effective modulation of the arachidonic acid metabolic cascade to counteract their inflammatory status.

Longevity is also characterized by decrease concentration of long chain triglycerides (TG 46:5, TG 47:5) and increase concentrations of very long TG chain with a high carbon number (TG 48:6, TG 52:2, TG 54:3). While usually highly unsaturated TG are target to peroxidation and the overall triglycerides family are seen as an adverse risk factor, recent investigations points to specific TG linked to adverse events where lipids of higher carbon number and double bond content were associated with decreased risk [48]. It is clear that these specific lipids in longevity remain to be determined as our centenarians present an overall net balance among increase/decrease TG species compared to elderly individuals. Lastly, we also noted that concentration of diacylglycerols decreases (DAG 26:0, DAG 26:1). DAG can result from the phosphatidic acid pathway, which represents the lipogenesis route in the synthesis of TAG and phospholipids. However intracellular DAGs can also be derived from TAG hydrolysis of lipid droplets, mediated by adipose triglyceride lipase (ATGL), and activation of phospholipase C, which will release DAGs from membrane lipids. Recent evidence support the hypothesis that increases in intracellular diacylglycerol content, due to an imbalance between fatty acid delivery and intracellular fatty acid oxidation and storage, leads to activation of new protein kinase C (PKC) isoforms that in turn inhibit insulin action in liver and skeletal muscle [49].

Although we tried to minimize the dietary influence by controlling time of sampling, it cannot be completely rejected, that some of the metabolism of some nutrient constituents and medications (as it is common for elderly) can affect the blood composition of our putative markers. Yet, our elderly and centenarian cohort represents a population from a restricted geographic area of Northern Italy and as such can be considered relatively homogeneous regarding lifestyle, dietary habits, and drug intake.

In conclusion, in this work we identified, by ^1^H-NMR metabonomics and shot-gun lipidomics, important metabolic signature in serum of healthy aging in a representative longevity cohort. It is important to denote that this work additionally complement previous investigations on longevity studies, yet specifically studying people who reached extreme longevity (centenarians). The represented changes reflect that longevity is marked by better antioxidant capacity and a well-developed membrane lipid remodelling process able to maintain cell integrity. Moreover, in the light of very recent data indicating glycerophosphocholine as a circulating marker related to cell senescence [30], our data are suggestive of the fact that centenarians are characterised by lower levels of cell senescence with respect to old subjects. As a whole, these data support the hypothesis that from a metabolic point of view centenarians are younger than their chronological age. Future work will be required to more precisely assess the predictive role of our markers in additional cohorts and to determine the effect of nutrition and genetic determinants in shaping healthy aging.

## METHODS

### Subjects and study groups

A total of 294 subjects belonging to two age groups were enrolled from four Italian cities (Bologna, Milan, Florence, Parma)(Table 1). The group of centenarians consisted of 98 subjects (mean age 100.7± 2.1 yrs) born in Italy between the years 1900 and 1908. The elderly group includes 196 subjects (mean age 70 ± 6 yrs). The study protocol was approved by the Ethical Committee of Sant'Orsola-Malpighi University Hospital (Bologna, Italy). ^1^H-NMR profiling was applied on 98 centenarians (average age 100.7, 22 males and 76 females) and 196 elderly (average age 70.4, 85 males and 111 females). Lipidomics was performed on 15 centenarians (average age 100.0, 3 males and 12 females) and 37 elderly (average age 69.7, 16 males and 21 females). Overnight fasting blood samples were obtained in the morning (between 7 and 8 a.m.). Serum was obtained after clotting and centrifugation at 760 g for 20 min at 4°C, and immediately frozen and stored at −80 °C. After obtaining written informed consent, a standard questionnaire was administered by trained physicians and nursing staff to collect demographic and lifestyle data, anthropometric measurements, functional, cognitive and health status, clinical anamnesis. Current use of medications follows procedure previously reported [15] protocol.

### Clinical chemistry

Overnight fasting blood samples were obtained early in the morning. Serum total and HDL cholesterol, triglycerides, CRP, insulin resistance (HOMA-IR) were determined using standard hematology methods.

### Sample preparation for ^1^H NMR Spectroscopy

40 μl serum samples were mixed with 20 μl D2O containing a buffer solution (KH_2_PO_4_, final concentration of 0.2 M). After centrifugation, samples were transferred into 1.7 mm diameter NMR tubes by using a syringe. Metabolic profiles were measured on a Bruker Avance III 600 MHz spectrometer at 300 K (Bruker Biospin, Rheinstetten, Germany). A standard Carr-Purcell-Meiboom-Gill (CPMG) spin-echo sequence with water suppression [50] spectra was acquired for each sample measured using a spin-echo loop time (2nτ) of 19.2 ms and a relaxation delay D1 of 2.5 s. For each spectrum 64 FIDs were collected into 98k data points using a spectral width of 30 ppm corresponding to an acquisition time of 2.7 s. Acquired ^1^H NMR spectra were processed using the Topspin software package (version 2.1; Bruker Biospin, Rheinstetten, Germany) and were referenced to the glucose at δ = 5.23. The peak assignment to specific metabolites was achieved using an internal library of compounds and the literature [51;52] and confirmed by standard two-dimensional NMR spectroscopy (JRES, TOCSY, HSQC, HMBC) on selected samples. For statistical analysis all NMR spectra were converted into 12 K data points over the range of δ 0.4-10.0 and imported into the MATLAB software (version 7.14.0 (R2012a; The MathWorks Inc., Natick, MA) excluding the water residue (water δ=4.67-4.97). The spectra were normalized to the total sum of all intensities within the specified range.

### Automated sample preparation for shot-gun lipidomics

A 96 samples high throughput, fully automated liquid/liquid extraction method utilizing a Hamilton Microlabstar robot (Hamilton, Bonaduz, Switzerland) was developed in house for lipidomics extraction with minor modifications from previous methods [53]. Briefly, 5μL of serum was used for delipidation. Lipid extraction was perfomed with 700 μL MTBE/MeOH (10/3) containing an internal standard mixture of 5μM TAG 44:1, 0.5μM DAG 24:0, 5μM PC 28:0, 1μM LPC 14:0, 1μM PE 28:0, 0.5μM LPE 14:0, 1μM PS 28:0, 0.5μM LPS 17:1, 1μM PI 32:0, 0.5μM LPI 17:1, 0.5μM PA 28:0, 0.5μM LPA 14:0, 1μM PG 28:0, 0.5μM LPG 14:0, 2μM SM 35:1, 1μM Cer 32:1. Samples were vortexed at 4°C for 1 hour, followed by the addition of 150μL water to induce phase separation. After centrifugation for 10 min at 5,000g, 500μL of the upper organic phase was transferred into a 96-deepwell-plate (Eppendorf, Hamburg, Germany), sealed with aluminum foil and stored at −20°C until analysis. Prior to MS analysis 10μL of total lipid extract were finally diluted with 90μL of MS running buffer (isopropanol/methanol/chloroform 4:2:1 (v/v/v) containing 7.5mM ammonium acetate).

### Identification and Quantification of lipid species in plasma

A shotgun lipidomics approach was developed in house based on previously published work [54]. Analysis was carried out on an LTQ Orbitrap Velos MS (Thermo Fisher Scientfic, Reinach, Switzerland) system coupled to a Nanomate nanoinfusion ion source (Advion Bioscience Ltd, Harlow, Essex, UK). For each sample extract, two consecutive injections were realized for negative and positive ionization mode, respectively. Centroided high collisional dissociation (HCD) negative MS/MS were acquired in DDA mode. Each DDA cycle consisted of one MS survey spectra acquired at the target resolution Rm/z400 of 100,000, followed by the acquisition of 20 HCD FT MS/MS spectra at the resolution Rm/z400 of 30,000. One DDA experiment was completed in 25 min. Precursor ions were subjected to MS/MS if their m/z matched the masses of a pre-compiled inclusion list with the accuracy of 5 ppm. For mass accuracy, we used the lock mass option, where each spectrum has been individually corrected for potential mass drifts. In positive ionization mode MS spectra were acquired at the target resolution Rm/z400 of 100,000, no further MS/MS experiments were performed. The lock mass option was enabled using LPA 17:0 (m/z 424.492; negative mode) and d18:1/17:0 Cer (m/z 551.528; positive mode) as reference peaks.

Lipid species were identified by LipidXplorer following the available protocol [55]. Data were then exported and further processed by an in-house developed software tool. The routine merged the data sets and generated Excel-output-files containing the normalized values (Internal standard to analyte ratio) and absolute concentrations by comparing the abundances of precursor ions of analyte and internal standard spiked prior to extraction.

### Chemicals and lipid standards

Ethanol, chloroform and iso-Propanol (HPLC grade) were purchased from Biosolve (Valkenswaard, the Netherlands). Methanol, water and Ammoniumacetate were obtained from Merck (Darmstadt, Germany). Synthetic lipid standards were purchased from Avanti Polar Lipids with purities higher than 99 %. Stock-solutions of individual lipid compounds were prepared in methanol and stored at −20°C. Working solutions of the desired concentration were prepared by dilution in isopropanol/methanol/ chloroform 4:2:1(v/v/v).

### Lipid nomenclature

Lipids have been named according to Lipid Maps (http://www.lipidmaps.org) with the following abbreviations: PC, Phosphatidylcholine; PC-O, Phsophatidylcholine-ether; LPC, Lysophosphati-dylcholine; PE, Phosphatidylethanolamine; PE-O, Phsophatidylethanolamine-ether; LPE, Lysophosphati-dylethanolamine; PS, Phosphatidylserine; LPS, Lysophosphatidylserine; PI, Phosphatidylinositol; LPI, Lysophosphatidylinositol; PG, Phosphatidylglycerol; Cer, Ceramide; SM, Sphingomyelin; DAG, Diacyl-glycerol; TAG, Triacylglycerol, Phosphatidic acid; PA.

Individual lipid species were annotated as follows: [lipid class] [total number of carbon atoms]:[total number of double bonds]. For example, PC 34:4 reflects a phosphatidylcholine species comprising 34 carbon atoms and 4 double bonds.

### Multivariate Data Analysis

Multivariate Data Analysis (MVA) was performed in several software environments. Thus, data import and pre-processing steps for both 1H NMR and targeted MS data were done using ‘in-house’ routines written in MATLAB (version 7.14.0, The Mathworks Inc., Natick, MA, USA) and R (R Core Team (2012). R: A language and environment for statistical computing. R Foundation for Statistical Computing, Vienna, Austria. ISBN 3-900051-07-0, URL http://www.R-project.org/). In NMR data analysis Orthogonal Projection on Latent Structures – discriminant analysis (OPLS-DA) models were carried out by using the SIMCA-P+ software (version 13.0, Umetrics AB, Umeå, Sweden). Targeted MS data was analyzed by Random Forests by using the package ‘randomForest’ running in the R environment. Univariate significance tests for were also performed in R using the package ‘stats’. A level of significance of 0.05 or less was considered significant.

## SUPPLEMENTARY FIGURES AND TABLES


