# Supplementary material for: Serum profiling of healthy aging identifies phospho- and sphingolipid species as markers of human longevity
Source: Aging (Albany NY). 2014 Jan 21;6(1):9–25. doi: 10.18632/aging.100630 (PMC3927806; doi:10.18632/aging.100630)
Supplement: Supplementary file 1 [file aging-06-009-s001.pdf]

SUPPLEMENTARY FIGURES AND TABLES

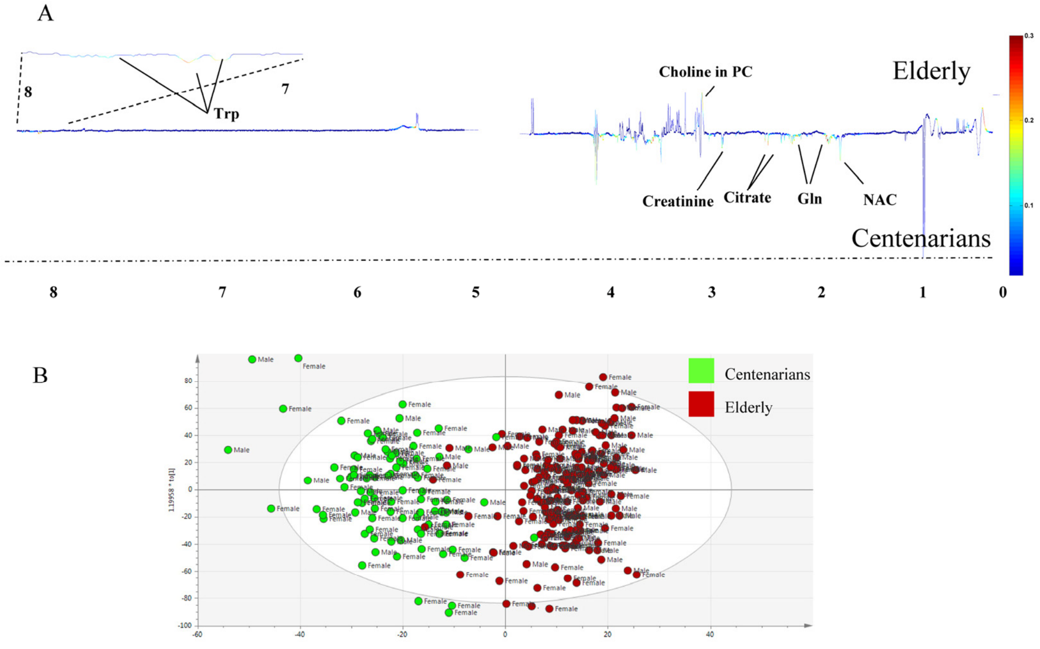

Figure S1. OPLS-DA coefficient (**A**) and score (**B**) plots derived from serum 1H-NMR spectra from elderly and centenarians.

Table S1. O-PLS-DA model summary for discriminating serum metabolic profiles

Overview	$R2X_{(cum)}$	$R2Y_{(cum)}$	Q^2Y	AuROC
Centenarians vs. Elderly	0.15	0.79	0.54	0.99

Table S2. All significantly regulated metabolites in both genders (mean values ± SD) from the ^{1}H-NMR profiling of elderly and centenarians serum. Significant differences were confirmed by paired t tests (2 tailed) and marked as follow: *p<0.05, **p<0.01, ***p<0.001. Orange color refers to increased concentration, blue color refers to decreased concentration.

Peak Integral (a.u.)	Chemical shift	Centenarians	Elderly
		Mean ± SD	Mean ± SD
NAC	2.04 (s)	2.08 ± 0.02***	1.94 ± 0.18
Glutamine	2.13 (m)	7.01 ± 1.61***	5.93 ± 1.07
PC in phoshatidylcholine	3.21 (s)	3.25 ± 0.41*	3.44 ± 0.61
Citrate	2.69 (d)	1.01 ± 0.02***	0.82 ± 1.36
Creatinine	3.05 (s)	1.73 ± 0.38***	1.44 ± 0.29
Phenylalanine	7.33 (m)	8.29 ± 1.76***	6.53 ± 1.36

Table S3. All significantly regulated metabolites in males in blood serum (mean values ± SD) from the ^{1}H-NMR profiling of elderly and centenarians. Significant differences were confirmed by paired t tests (2 tailed) and marked as follow: *p<0.05, **p<0.01, ***p<0.001. Orange color refers to increased concentration; blue color refers to decreased concentration.

Peak Integral (a.u.) *Males*	Chemical shift	Centenarians	Elderly
		Mean ± SD	Mean ± SD
NAC	2.04 (s)	2.09 ± 0.02***	1.95 ± 0.17
Glutamine	2.13 (m)	6.98 ± 1.41***	6.13 ± 1.41
PC in phoshatidylcholine	3.21 (s)	3.17 ± 0.48*	3.31 ± 0.67
Citrate	2.69 (d)	1.01 ± 0.02***	0.83 ± 1.36
Creatinine	3.05 (s)	1.79 ± 0.41***	1.54 ± 0.29
Phenylalanine	7.33 (m)	8.48 ± 1.85***	6.59 ± 1.35

Table S4. All significantly regulated metabolites in females in blood serum (mean values ± SD) from the ^{1}H-NMR profiling of elderly and centenarians. Significant differences were confirmed by paired t tests (2 tailed) and marked as follow: *p<0.05, **p<0.01, ***p<0.001. Orange color refers to increased concentration; blue color refers to decreased concentration.

Peak Integral (a.u.) *Females*	Chemical shift	Centenarians	Elderly
		Mean ± SD	Mean ± SD
NAC	2.04 (s)	2.07 ± 0.02***	1.92 ± 0.19
Glutamine	2.13 (m)	7.01 ± 1.33***	5.93 ± 1.07
PC in phoshatidylcholine	3.21 (s)	3.27 ± 0.61*	3.53 ± 0.69
Citrate	2.69 (d)	1.00 ± 0.02***	0.81 ± 1.38
Creatinine	3.05 (s)	1.71 ± 0.36***	1.44 ± 0.29
Phenylalanine	7.33 (m)	8.23 ± 1.72***	6.48 ± 1.37

Table S5. All significantly regulated metabolites in both genders (mean values ± SD) from the shot gun lipidomics approach on the elderly and centenarians. Significant differences were were confirmed by paired t tests (2 tailed) and marked as follows: *p<0.05, **p<0.01,***p<0.001. Orange color refers to increased concentration; blue color refers to decreased concentration.

**Lipid species**[μM/l]	**Centenarians**	**Elderly**
	Mean ± SD	Mean ± SD
Cer 42:2	2.35 ± 0.76	0.42 ± 0.39***
DAG 26:0	0.26 ± 0.66	3.41 ± 1.19***
DAG 26:1	0.32 ± 0.87	2.68 ± 0.85***
LPC 18:1	24.2 ± 3.53	16.8 ± 5.02***
PC 14:0/18:1	23.4 ± 8.79	7.04 ± 4.83***
PC 16.0/18.1	351 ± 61.3	168 ± 59.2***
PC 16.0/18.2	599 ± 140	392 ± 114***
PC 16.0/18.3	13.6 ± 4.6	3.68 ± 3.65***
PC 18.0/22.5	12.3 ± 3.82	2.26 ± 2.56***
PC-O 28:0	19.5 ± 3.02	47.6 ± 8.96***
PC-O 30:0	34.5 ± 3.20	78.7 ± 13.3***
PC-O 32:1	2.04 ± 1.65	0.11 ± 0.45***
PC-O 34:1	8.02 ± 2.44	0.81 ± 1.50***
PC-O 34:2	8.52 ± 3.16	2.01 ± 2.60***
PC-O 36:3	4.11 ± 2.62	0.07 ± 0.42***
PC-O 38:4	8.15 ± 2.94	1.22 ± 1.80***
PC-O 38.5	22.1 ± 4.65	9.94 ± 5.02***
PC-O 38.6	4.91 ± 3.37	0.37 ± 1.27***
PE 16:0/20:4	1.77 ± 1.19	0.139± 0.50*
PE 18:0/20:2	2.02 ± 1.05	0.221± 0.42***
PE 18:0/20:3	1.50 ± 0.81	0.08± 0.28***
PE 18:0/20:4	7.84 ± 2.43	3.83 ± 2.25***
PE 18:2/18:0	7.87 ± 1.92	3.32 ± 1.99***
PI 18:0/18:1	2.37 ± 0.97	0.68 ± 0.65***
PI 18:1/16:0	2.97 ± 1.17	0.80 ± 0.63***
PI 20.3/18:0	5.55 ± 0.66	2.62 ± 1.20***
SM 33:1	11.9 ± 2.43	6.09 ± 2.88***
SM 34:1	150 ± 21.3	99.2 ± 23.7***
SM 36:1	25.1 ± 5.04	16.5 ± 4.97***
SM 36:2	10.8 ± 3.16	5.56 ± 3.42*
SM 38:2	5.54 ± 2.26	1.29 ± 1.23***
SM 41:2	14.6 ± 3.67	7.92 ± 3.96*
SM 42:2	72.5 ± 12.2	44.2 ± 11.1***
SM 42:3	35.8 ± 7.04	20.2 ± 7.04***
SM 42:4	1.63 ± 1.17	0.05 ± 0.30*
SM 50:1	3.95 ± 0.86	7.30 ± 2.00***
TAG 46:5	10.8 ± 3.58	18.4 ± 6.45**
TAG 47:5	3.167 ± 2.72	7.53 ± 2.57**
TAG 48:6	13.3 ± 3.09	7.38 ± 7.84*
TAG 52:2	109.9 ± 34.4	57.0 ± 27.3***
TAG 54:3	32.7 ± 13.198	15.3 ± 9.63***

Table S6. All significantly regulated metabolites in males (mean values ± SD) from the shot gun lipidomics approach on the elderly and centenarians. Significant differences were assessed by paired t tests (2 tailed) and marked as follows: *p<0.05, **p<0.01, ***p<0.001. Orange color refers to increased concentration; blue color refers to decreased concentration.

**Lipid species**[μM/l] *Males*	**Centenarians** Mean ± SD	**Elderly** Mean ± SD
Cer 42:2	2.56 ± 0.17	0.41 ± 0.59***
DAG 26:0	0.590 ± 0.83	3.29 ± 1.64***
DAG 26:1	0.49 ± 0.69	2.61 ± 1.25***
LPC 18:1	27.3 ± 3.59	18.1 ± 8.21***
PC 14:0/18:1	23.45 ± 0.63	5.88 ± 4.28***
PC 16.0/18.1	327.9 ± 58.1	165.1 ± 53.9***
PC 16.0/18.2	625.5 ± 151.2	370.6 ± 131.1***
PC 16.0/18.3	14.4 ± 3.12	3.32 ± 3.78***
PC 18.0/22.5	12.0 ± 2.53	2.31 ± 3.21***
PC-O 28:0	19.5 ± 1.34	45.6 ± 13.3***
PC-O/30:0	35.6 ± 2.36	74.7 ± 20.8***
PC-O 32:1	2.51 ± 1.83	0.31 ± 0.71***
PC-O 34:1	9.23 ± 2.24	1.11 ± 2.21***
PC-O 34:2	8.81 ± 2.25	2.95 ± 3.44***
PC-O 36:3	4.35 ± 1.32	0.43 ± 1.19***
PC-O 38:4	9.71 ± 4.34	2.00 ± 1.17***
PC-O 38.5	23.6 ± 6.31	10.5 ± 5.08***
PC-O 38.6	4.73 ± 3.55	0.94 ± 1.98*
PE 16:0/20:4	1.51 ± 1.33	0.06 ± 0.51*
PE 18:0/20:2	1.68 ± 0.57	0.28 ± 0.61*
PE 18:0/20:3	1.12 ± 0.02	0.15 ± 0.04*
PE 18:0/20:4	6.48 ± 1.15	2.93 ± 1.94**
PE 18:2/18:0	7.53 ± 1.58	2.82 ± 1.98*
PI 18:0/18:1	2.378 ± 0.973	0.688 ± 0.657*
PI 18:1/16:0	1.60 ± 0.56	0.72 ± 0.81*
PI 20.3/18:0	5.71 ± 0.09	2.51 ± 1.29*
SM 33:1	12.2 ± 2.46	5.61 ± 3.15*
SM 34:1	151 ± 30.5	94.8 ± 25.8**
SM 36:1	22.9 ± 4.81	15.4 ± 5.86*
SM 36:2	8.71 ± 2.19	4.52 ± 3.69*
SM 38:2	4.45 ± 1.62	1.05 ± 1.19*
SM 41:2	14.09 ± 3.94	7.39 ± 3.21**
SM 42:2	75.7 ± 15.8	44.1 ± 12.1***
SM 42:3	35.2 ± 7.31	19.3 ± 7.31**
SM 42:4	1.22 ± 0.91	0.08± 0.31*
SM 50:1	4.29 ± 1.31	6.79 ± 2.15*
TAG 46:5	10.14 ± 3.58	17.0 ± 3.47**
TAG 47:5	2.86 ± 2.07	6.60 ± 2.84**
TAG 48:6	11.7 ± 1.04	6.73 ± 4.24**
TAG 52:2	85.7 ± 10.0	51.5 ± 20.6**
TAG 54:3	22.8 ± 1.27	14.4 ± 10.5*

Table S7. All significantly regulated metabolites in females (mean values ± SD) from the shot gun lipidomics approach on the elderly and centenarians. Significant differences were confirmed by paired t tests (2 tailed) and young and marked as follows: *p<0.05, **p<0.01, ***p<0.001. Orange color refers to increased concentration; blue color refers to decreased concentration**.**

**Lipid species**[μM/l] *Females*	**Centenarians** Mean ± SD	**Elderly** Mean ± SD
Cer 42:2	2.11± 1.00	0.65 ± 0.55*
DAG 26:0	0.51 ± 1.23	3.08 ± 1.16**
DAG 26:1	0.53 ± 1.15	2.42 ± 0.81*
LPC 18:1	22.9 ± 6.29	18.1 ± 4.59*
PC 14:0/18:1	22.1 ± 8.91	8.84 ± 4.99*
PC 16.0/18.1	342 ± 75.6	189.3 ± 71.7***
PC 16.0/18.2	557 ± 168	442 ± 114***
PC 16.0/18.3	12.3 ±5.83	5.23 ± 4.34***
PC 18.0/22.5	11.4 ± 5.1	3.63 ± 2.61***
PC-O 28:0	21.7 ± 8.07	44.8 ± 10.8***
PC-O 30:0	37.3 ± 10.1	75.3 ± 15.1***
PC-O 32:1	1.74 ± 1.53	0.07 ± 0.31*
PC-O 34:1	7.04 ± 3.04	1.41 ± 2.09***
PC-O 34:2	7.72 ± 3.87	2.27 ± 2.81***
PC-O 36:3	3.71 ± 2.86	0.55 ± 1.82***
PC-O 38:4	7.08 ± 2.81	1.86 ± 3.01***
PC-O 38.5	19.94 ± 6.99	11.3 ± 5.84***
PC-O 38.6	4.55 ± 3.32	0.91 ± 1.09***
PE 16:0/20:4	1.70 ± 1.16	0.22 ± 0.61*
PE 18:0/20:2	2.02 ± 1.05	0.22 ± 0.42**
PE 18:0/20:3	1.47 ± 0.81	0.23 ± 0.21*
PE 18:0/20:4	7.71 ± 2.71	4.96 ± 2.16**
PE 18:2/18:0	7.37 ± 2.61	4.28 ± 2.14*
PI 18:0/18:1	2.37 ± 1.12	0.93 ± 0.79**
PI 18:1/16:0	2.86 ± 1.34	1.15 ± 0.66*
PI 20.3/18:0	5.24 ± 1.11	3.17 ± 1.54*
SM 33:1	10.9 ± 3.96	7.41 ± 3.18*
SM 34:1	141 ± 30.6	108.8 ± 23.5**
SM 36:1	24.1 ± 7.09	19.2 ± 5.22*
SM 36:2	10.5 ± 4.26	7.55 ± 3.71*
SM 38:2	5.35 ± 2.67	2.28 ± 2.36*
SM 41:2	13.5 ± 5.22	9.81 ± 4.52*
SM 42:2	67.5 ± 16.9	46.6 ± 11.0*
SM 42:3	33.3 ± 10.1	22.4 ± 7.22*
SM 42:4	1.59 ± 1.21	0.08 ± 0.37*
SM 50:1	4.01 ± 0.87	7.19 ± 2.00*
TAG 46:5	11.4 ± 3.96	18.5 ± 8.61*
TAG 47:5	3.62 ± 2.90	7.39 ± 3.05*
TAG 48:6	12.6 ± 4.88	9.01 ± 7.81*
TAG 52:2	110.3 ± 38.6	66.5 ± 28.3***
TAG 54:3	32.6 ± 15.1	18.5 ± 10.8***
